# A cost-effectiveness analysis of maternal and neonatal health interventions in Ethiopia

**DOI:** 10.1093/heapol/czz034

**Published:** 2019-05-18

**Authors:** Solomon Tessema Memirie, Mieraf Taddesse Tolla, Dawit Desalegn, Mengistu Hailemariam, Ole Frithjof Norheim, Stéphane Verguet, Kjell Arne Johansson

**Affiliations:** 1 Department of Pediatrics and Child Health, College of Health Sciences, Addis Ababa University, Addis Ababa, Ethiopia; 2 Department of Global Public Health and Primary Care, University of Bergen, N Bergen, Norway; 3 Department of Global Health and Population, Harvard T.H. Chan School of Public Health, Boston, MA, USA; 4 Department of Gynecology and Obstetrics, Addis Ababa University, Addis Ababa, Ethiopia; 5 International Reproductive Health Training Center, Addis Ababa, Ethiopia

**Keywords:** Maternal and neonatal health, cost-effectiveness analysis, Ethiopia

## Abstract

Ethiopia is one of the sub-Saharan African countries contributing to the highest number of maternal and neonatal deaths. Coverage of maternal and neonatal health (MNH) interventions has remained very low in Ethiopia. We examined the cost-effectiveness of selected MNH interventions in an Ethiopian setting. We analysed 13 case management and preventive MNH interventions. For all interventions, we used an ingredients-based approach for cost estimation. We employed a static life table model to estimate the health impact of a 20% increase in intervention coverage relative to the baseline. We used disability-adjusted life years (DALYs) as the health outcome measure while costs were expressed in 2018 US$. Analyses were based on local epidemiological, demographic and cost data when available. Our finding shows that 12 out of the 13 interventions included in our analysis were highly cost-effective. Interventions targeting newborns such as neonatal resuscitation (institutional), kangaroo mother care and management of newborn sepsis with injectable antibiotics were the most cost-effective interventions with incremental cost-effectiveness ratios of US$7, US$8 and US$17 per DALY averted, respectively. Obstetric interventions (induction of labour, active management of third stage of labour, management of pre-eclampsia/eclampsia and maternal sepsis, syphilis treatment and tetanus toxoid during pregnancy) and safe abortion cost between US$100 and US$300 per DALY averted. Calcium supplementation for pre-eclampsia and eclampsia prevention was the least cost-effective, with a cost per DALY of about US$3100. Many of the MNH interventions analysed were highly cost-effective, and this evidence can inform the ongoing essential health services package revision in Ethiopia. Our analysis also shows that calcium supplementation does not appear to be cost-effective in our setting.

**Key Messages**

- Most neonatal and obstetric interventions are highly cost-effective in an Ethiopian setting.
- Neonatal resuscitation and kangaroo mother care were the most cost-effective interventions while calcium supplementation was the least cost-effective among the 13 interventions included in our analysis.
- The evidence can inform the ongoing essential health services package revision in Ethiopia.

## Introduction

With 11 000 women dying during pregnancy or childbirth, Ethiopia is one among the 10 countries that contribute to 60% of the global maternal deaths estimated in 2015 (Trends in maternal mortality: 1990 to 2015, 2015). Even though Ethiopia has witnessed a significant decline in under-five mortality (Central Statistical Agency (CSA) [Ethiopia] and ICF, 2016); the proportion of under-five deaths that occur during the first 28 days of life is increasing. Neonatal deaths accounted for 30% and 43% of under-five deaths in the 2000 and 2016 Demographic and Health Surveys (DHS), respectively (Central Statistical Agency (CSA) [Ethiopia] and ICF, 2001, 2016). Ethiopia is among the five sub-Saharan African countries contributing to the highest number of neonatal deaths, with >90 000 deaths in 2016 (Levels and Trends in Child Mortality, 2017).

Maternal and neonatal deaths arise from the risks attributable to pregnancy and childbirth as well as from low coverage and poor-quality health services (Freedman *et al.*, 2005). Effective interventions during the antenatal period, the time around birth, and the first week of life that can significantly decrease maternal and neonatal deaths are available (Trends in maternal mortality: 1990 to 2015, 2015; Levels and Trends in Child Mortality, 2017), but their coverage levels have remained very low in Ethiopia: coverage of skilled birth attendance and post-natal visits (within 2 days of birth), were in 2015 28% and 13%, respectively (Central Statistical Agency (CSA) [Ethiopia] and ICF, 2016).

Maternal and child health has been the focus of both the Millennium Development Goals and sustainable development goals (SDGs) (Sustainable Development Knowledge Platform, 2018). SDG targets 3.1 and 3.2 for maternal and child mortality represent a transformative new agenda towards ending preventable maternal and child deaths globally; all countries aiming to reduce maternal deaths to 70 per 100 000 live births and neonatal mortality to 12 per 1000 live births by 2030 (Sustainable Development Knowledge Platform, 2018). The Ministry of Health of Ethiopia intends to achieve population health levels commensurate with the average of lower middle-income countries in 2025 and upper middle-income countries in 2035 (Admasu *et al.*, 2014). The national targets set are in line with the SDGs targets for maternal and neonatal deaths. Achievement of these targets requires scale-up of high impact and cost-effective maternal and neonatal interventions in Ethiopia. This is particularly important given the resource scarcity in Ethiopia as evidenced by the sixth National Health Accounts, which reported Ethiopia’s total health expenditure per capita at about US$30 for the years 2013/14 (Federal Democratic Republic of Ethiopia Ministry of Health, 2017).

Cost-effectiveness analysis (CEA) in the evaluation of health delivery has become an increasingly accepted component of health policy and planning (World Health Organization, 2010). Experience from several countries, however, has shown that CEA results are rarely used to inform policy decision-making largely due to lack of contextualized evidence (Tan-Torres Edejer *et al.*, 2003). Absence of local data on estimates of cost and effectiveness of health interventions leads health planners to use evidence generated elsewhere, often from a setting different to the local one. However, studies have shown that several factors may alter the actual cost-effectiveness of a given intervention across settings (Hutubessy *et al.*, 2003). Evidence on cost-effectiveness of interventions to improve maternal and neonatal health (MNH) outcomes in developing countries is scarce and to our knowledge non-existent in Ethiopia.

In this article, we examine the cost-effectiveness of selected MNH interventions in an Ethiopian setting. The evidence generated could inform policymakers on how to efficiently use scarce financial resources.

## Methods

### Interventions

We evaluate 13 MNH interventions that are provided during pregnancy, childbirth and the neonatal period (Table 1). Interventions were selected based on expert recommendations and scientific evidence of benefit to maternal and neonatal survival (Blencowe *et al.*, 2010; Cousens *et al.*, 2010; Lawn *et al.*, 2010; Mwansa-Kambafwile *et al.*, 2010; Blencowe *et al.*, 2011; Hussain *et al.*, 2011; Imdad *et al.*, 2011; Jabeen *et al.*, 2011; Lee *et al.*, 2011; Pollard *et al.*, 2013; Ronsmans and Campbell, 2011; Zaidi *et al.*, 2011). The interventions included were: safe abortion; interventions during pregnancy [tetanus toxoid, syphilis detection and treatment, calcium supplementation, management of pre-eclampsia and eclampsia,^1^ antibiotics for preterm pre-labour rupture of membrane (pPRoM), antenatal corticosteroids for preterm labour, induction of labour]; intrapartum interventions (active management of the third stage of labour); post-partum interventions (maternal sepsis case management, newborn sepsis management, neonatal resuscitation and kangaroo mother care). These interventions were selected because they had the most data available, target the most important maternal and neonatal disease conditions in Ethiopia and were among the national priorities of the Ethiopia’s ministry of health (Federal Democratic Republic of Ethiopia Ministry of Health, 2015). Most of these interventions, except calcium supplementation, are part of the essential services rendered in public health facilities in Ethiopia though their coverage is very low (Table 1). For safe abortion services and tetanus toxoid baseline coverage data were extracted from the Health Sector Transformation Plan and the 2016 Ethiopia Health and Demographic Survey, respectively (Federal Democratic Republic of Ethiopia Ministry of Health, 2015; Central Statistical Agency [CSA] [Ethiopia] and ICF, 2016), but for the other interventions, we used baseline coverage rates from the Lives Saved Tool (LiST) (Avenir Health, 2017). Given the low coverage of interventions, we set modest target coverage with a 20 percentage points increase from the baseline of all the interventions.

**Table 1:** Interventions included in the analysis, their description, target population, baseline and target coverage rates

Intervention	Description of the intervention (number of visits)	Population in need	Baseline coverage (2017), %	Target coverage (Post 2017),^a^ %
Safe abortion	Medical abortion using 800 μg vaginal misoprostol^b^ (two visits)	Women seeking abortion (20% of births)	37	57
Tetanus toxoid	Two tetanus toxoid injections^b^ (two visits)	All pregnant women during ANC visit	49	69
Syphilis detection and treatment	Screening of all pregnant women by RPR test and treatment of identified cases with benzathine penicillin^b^ (one visit)	All pregnant women during ANC visit	31	51
Calcium supplementation	Routine calcium supplementation during pregnancy^b^ (one visit)	All pregnant women during ANC visit	0	20
Management of pre-eclampsia and eclampsia	Package of care including anti-hypertensives and magnesium sulfates^b^ (three hospital bed days)	Women with pre-eclampsia (2.8% of births) and eclampsia (1%)	3	23
Antibiotics for pPRoM	Administration of oral antibiotics to women with pPRoM^b^ (two hospital bed days)	Women with pPRoM (4% of births)	3	23
Antenatal corticosteroids for preterm labour	Administration of steroids and inpatient care of women with suspected preterm labour^b^ (two hospital bed days)	Women with premature labour (10.1% of births)	0	20
Active management of the third stage of labour	Administration of prophylactic oxytocin, cord clamping and delivery of the placenta by controlled cord traction^b^	All pregnant women	23	43
Induction of labour (beyond 41 weeks)	Induction of labour at 41 weeks gestation with 200 μg misoprostol^b^	Women at gestational age 41+ weeks (5% of births)	3	23
Maternal sepsis case management	Development of sepsis within the 42 days following delivery, requiring inpatient care including treatment with antibiotics^b^ (four hospital bed days)	Women with infection within 42 days of giving birth (4.1% of births)	22	42
Neonatal resuscitation (institutional)	Detection of breathing problems and resuscitation if required	Births with asphyxia (1% of births)	26	46
Newborn sepsis	Administration of IV/IM antibiotics for neonatal sepsis, meningitis, or pneumonia^b^	Neonates with infection (10% of births)	26	46
Kangaroo mother care	Thermal care for newborn babies weighing <2000 g through continual skin to skin contact (1 visit)	Low birth weight babies (15% of births)	22	42

a Target coverage is an increase in 20% coverage points from the baseline.

b Details of the drug regimen, other supplies and laboratory cost are provided in Web Appendix I.

IV/IM, intravenous/intramuscular; pPRoM, preterm pre-labour rupture of membrane; RPR, rapid plasma reagin.

### Intervention effectiveness and health impacts

Efficacy data were based on available evidence in accordance with a recent update for LiST (Blencowe *et al.*, 2010; Cousens *et al.*, 2010; Lawn *et al.*, 2010; Mwansa-Kambafwile *et al.*, 2010; Blencowe *et al.*, 2011; Hussain *et al.*, 2011; Imdad *et al.*, 2011; Jabeen *et al.*, 2011; Lee *et al.*, 2011; Ronsmans and Campbell, 2011; Zaidi *et al.*, 2011; Pollard *et al.*, 2013). We used LiST to determine cause-specific neonatal and maternal deaths associated with diseases/conditions for the baseline coverage levels. LiST combines national inputs on age-specific population size, disease incidence, prevalence and mortality to calculate the number of maternal and neonatal lives saved for a given set of interventions at a specified increase in coverage (Boschi-Pinto *et al.*, 2010). The number of cause-specific deaths that would be prevented for a change in the coverage of a given intervention would be calculated as (Winfrey *et al.*, 2011):

(1) $$\mathrm{Deaths}\;\mathrm{prevented}=\;\frac{N*I*\left(P1-P0\right)*\mathrm{AF}}{\left(1-I*P0\right)},$$

where, *N* is the number of deaths at baseline coverage, *I* is the intervention effectiveness, *P*0 is the baseline intervention coverage, *P*1 is the intervention target coverage, AF is the attributable fraction (fraction of the adverse condition such as death that is attributable to a specific exposure). Table 2 presents cause-specific neonatal and maternal outcomes, related baseline deaths, attributable fractions and intervention effectiveness. Once the number of deaths prevented by each intervention was estimated, the disability-adjusted life years (DALYs) averted were also computed, based on the life table for Ethiopia adjusted by health state valuations from the World Health Organization cost-effectiveness and strategic planning (WHO-CHOICE) data for the African region (World Health Organization, 2018a and 2018b). DALYs averted by each intervention were then calculated as the sum of deaths preventable at each age, multiplied by disability-adjusted life expectancy at that age, which were discounted at 3% per year (Tan-Torres Edejer *et al.*, 2003).

**Table 2:** Intervention effectiveness on neonatal and maternal mortality and attributable fractions (of deaths)

Intervention	Neonatal outcome (number of deaths at baseline coverage)	Risk reduction on neonatal mortality (%)	Attributable fraction	Maternal outcome (number of deaths at baseline coverage)	Risk reduction on maternal mortality (%)	Attributable fraction
Safe abortion				Deaths from unsafe abortion (905)	95	1
Tetanus toxoid (pregnant women)	Deaths from tetanus (1684)	94	1	Deaths from indirect causes (2887)	98	0.0125
Syphilis detection and treatment (pregnant women)	Deaths from severe infection (18 168)	97	0.04			
	Deaths from congenital abnormality (10 227)	80	0.027			
Calcium supplementation				Deaths from hypertensive disorders during pregnancy (1734)	20	1
Management of pre-eclampsia and eclampsia				Deaths from hypertensive disorders during pregnancy (1734)	59	1
Antibiotics for pPRoM	Deaths from neonatal sepsis (12 292)	39	0.198			
	Deaths from prematurity (15 450)	12	0.33			
Antenatal corticosteroids for preterm labour	Deaths from prematurity (15 450)	31	0.33			
Active management of the third stage of labour				Deaths from post-partum haemorrhage (1716)	70	1
Induction of labour (beyond 41 weeks)	Deaths from asphyxia (22 611)	69	0.03			
Maternal sepsis case management				Deaths from sepsis (1171)	80	1
Neonatal resuscitation (institutional)	Deaths from asphyxia (22 611)	40	1			
Newborn sepsis—injectable antibiotics	Deaths from neonatal sepsis (12 292)	65	1			
	Deaths from neonatal pneumonia (5876)	75	1			
Kangaroo mother care	Deaths from prematurity (15 450)	40	1			

### Costs

Costs are estimated from the provider’s perspective and only direct medical costs borne by the provider at the point of service delivery were included. Costs were divided into patient- and programme-level costs. Patient-level costs included: drugs, supplies, laboratory, outpatient visits, inpatient stays and individual health education messages. Programme-level costs included resources to establish and maintain an intervention: administration, publicity, training and delivery of supplies.

To calculate costs, we used an ingredients approach that requires information on the quantities of inputs needed and their unit price (details of cost computation are presented in Supplementary Data). Costs were computed by multiplying quantities of inputs with their unit price. The quantities of resources used for the interventions were based on WHO-CHOICE assumptions (Table 1). Unit costs for hospital and health centre visits and hospital bed-day costs were extracted from WHO-CHOICE (World Health Organization, 2018c). Costs for laboratory tests were collected from several government health facilities. We used the average value of the laboratory cost depending on the level of service delivery. For drugs and supplies that are traded internationally, we used the median supplier price available internationally in 2015 (Table 3) with adjustment to include transportation costs (Joint Medical Store, 2014; Management Science for Health, 2016). We estimated programme-level costs to be 10% of patient-level costs, based on a previous study in Ethiopia (Mathewos *et al.*, 2017). We used the same programme-level costs across interventions, which is 10% of the average patient-level cost of all interventions included in our analysis. Direct non-medical costs (e.g. transportation) and indirect costs to patients and caregivers such as lost productivity were not included in the analysis. Total cost was the sum of patient- and programme-level costs. Costs were reported in 2018 US$.

**Table 3:** Unit cost inputs and socio-demographic data

Description	Unit prices, median (US$)	Description		Unit prices, median (US$)
Drugs and supplies (source: Management Science for Health, 2016)				
Misoprostol 200 µg (TAB/CAP)	0.1810	Amoxicillin suspension, 1 BOTT		0.4600
Paracetamol, 500 mg (TAB/CAP)	0.0044	Betamethasone 4 mg/ml (INJ)		0.2503
Tetanus toxoid vial (INJ) per dose	0.6869	Oxytocin 10 IU AMP (INJ)		0.1557
Benzanthine Pn 2.4 MU vial (INJ)	0.2612	Ampicillin 500 mg vial (INJ)		0.1507
Calcium Lactate 300 mg (TAB/CAP)	0.0129	Gentamicin 80 mg AMP (INJ)		0.0600
Sodium lactate solution, 500 ml (IV)	0. 5000	Gentamicin 20 mg AMP (INJ)		0.1760
MgSO4 5 g/10 ml vial (INJ)	0.7340	Metronidazole 500 mg vial (INJ)		0.5000
Lidocaine 2 ml AMP (INJ)	0.0972	Ceftriaxone 250 mg vial (INJ)		0.3538
Hydralazine, 20 mg AMP (INJ)	3.8578	Foley catheter, ch 14^a^		0.5662
Erythromycin 250 mg (TAB/CAP)	0.0306	Urine bag, 2 L^a^		0.3700
Amoxicillin 500 mg (TAB/CAP)	0.0300	Infant Bag and Mask^b^		20.44
Syphilis RPR (DIAG) per test	0.1250			
Healtd service delivery costs (source: World Health Organization, 2018c)				
Hospital (cost per visit)		1.59	Health centre (cost per visit)	1.96
Hospital (cost per bed day)		4.04		
Laboratory tests and procedures				
Complete blood count		1.98	CSF^c^	8.42
Blood culture and sensitivity		1.5		
Socio-demographic data				
Total population in 2018		107.5 million		United Nations Population Division (2017)
Live births in 2018		3.34 million		FMoH (2017) ^d^
GDP per capita ($US)		707		The World Bank (2016)
GDP per capita, PPP (Int.$)		1735		The World Bank (2016)
Official exchange rate (ETB per $US)		27.5		NBE (2018) ^e^

a *Source:* Joint Medical Store (2014).

b *Source:* https://www.laerdal.com/no/Search.aspx? q=Bag+and+Mask.

c Includes the cost of lumbar puncture, cerebrospinal fluid analysis, culture and sensitivity.

d *Source:* The Government of Federal Democratic Republic of Ethiopia (2017).

e *Source:* National Bank of Ethiopia (2018).

### Estimation of cost-effectiveness

We assessed the cost and health effects on the population of the interventions scale-up compared with the current coverage scenario. We opted to use the current coverage levels for the following reasons: (1) most of the interventions were part of the health services package though their coverage rates were very low, ranging from 0% to 49%; (2) For the current coverage levels, baseline mortality data were available from the LiST tool; (3) The current coverage level could impact the magnitude of incremental health benefits of intervention scale-up and subsequently the incremental cost-effectiveness ratios (ICERs); and (4) The policy relevance is likely higher when ICERs are estimated from the current coverage rates. ICERs were estimated dividing the incremental cost by incremental health effects of each intervention for the 20 percentage points coverage increase. Comparing the cost of the intervention with the gain in health could then be used to identify the most cost-effective sets of interventions. ICERs are reported in US$ per DALY averted for the year 2018.

### Uncertainty analysis

Given the uncertainty surrounding intervention costs and effectiveness inputs, we conducted a probabilistic uncertainty analysis with Monte Carlo simulations to assess the robustness of our findings (Baltussen *et al.*, 2002). We used a truncated normal distribution for both costs and effects with 15–25% standard deviations and proceeded to *n *=* *1000 simulation runs. We also conducted one-way sensitivity analyses where we varied the inputs as follows: applying 50% of the effectiveness estimates; doubling the price of drugs, supplies and laboratory tests; no discounting of health benefits; and 75% adherence to treatment.

## Results

Annual intervention costs, DALYs averted, and ICERs associated with a 20% coverage increase are shown in Table 4. Kangaroo mother care, neonatal resuscitation (institutional), antibiotics for preterm pre-labour rupture of membrane, antenatal corticosteroids for preterm labour and injectable antibiotics for newborn sepsis cost <US$100 per DALY averted. Induction of labour, safe abortion, management of pre-eclampsia/eclampsia, maternal sepsis case management, syphilis detection and treatment during pregnancy, active management of third stage of labour and tetanus toxoid during pregnancy cost between US$100 and US$300 per DALY averted. The ICER for Calcium supplementation is 3100 per DALY averted, which is 4.4 times the gross domestic product (GDP) per capita in Ethiopia. For all other interventions, the ICERs are less than US$300 per DALY averted, which is <0.4 times the GDP per capita in Ethiopia. The ICERs suggest that interventions for newborn care are highly cost-effective. Neonatal resuscitation, Kangaroo mother care and newborn sepsis management are all estimated to provide 1 DALY averted for a cost of less than US$20.

**Table 4:** Cost, effects and cost-effectiveness for a 20% points increase in coverage of maternal and neonatal interventions in Ethiopia in 2018

No.	Intervention	Total cost US$ (millions)	DALYs averted (millions)		ICER (US$ per DALYs averted)
			Discounted	Undiscounted	
1	Kangaroo mother care	0.29	0.037	0.074	8
2	Neonatal resuscitation (institutional)	0.36	0.055	0.110	7
3	Induction of labour (beyond 41 weeks)	0.39	0.003	0.005	152
4	Management of pre-eclampsia and eclampsia	0.52	0.005	0.008	108
5	Antibiotics for preterm pre-labour rupture of membrane (pPRoM)	0.59	0.009	0.017	69
6	Safe abortion	0.74	0.007	0.011	108
7	Antenatal corticosteroids for preterm labour	0.84	0.009	0.017	98
8	Newborn sepsis—injectable antibiotics	0.91	0.052	0.105	17
9	Maternal sepsis case management	1.15	0.005	0.009	220
10	Syphilis detection and treatment (pregnant women)	1.52	0.007	0.014	224
11	Active management of the third stage of labour	1.62	0.007	0.011	245
12	Tetanus toxoid (pregnant women)	2.69	0.016	0.032	168
13	Calcium supplementation	4.95	0.002	0.003	3081

Implementation of all 12 individual interventions (except calcium supplementation) for a 20% point increase in coverage would avert nearly 8000 neonatal deaths (11% reduction from baseline) and >1000 maternal deaths (10% reduction from baseline) at the cost of nearly US$12 million annually. Neonatal resuscitation, newborn sepsis management and kangaroo mother care would contribute to 80% of the neonatal deaths averted (nearly 6400 deaths) at a cost of nearly US$1.6 million.

**Figure 1:**
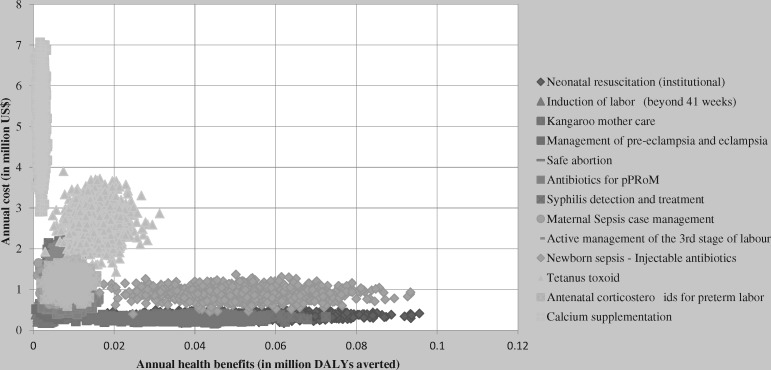
Probabilistic sensitivity analysis of MNH interventions in Ethiopia.

Table 5 summarizes the results of the one-way sensitivity analyses of individual interventions and shows the substantial uncertainty that resides within the cost-effectiveness estimates. Including undiscounted health benefits halves the ICER of all interventions making all interventions more cost-effective. However, decreasing the intervention effectiveness by 50% doubles the ICERs of interventions. Doubling the cost of drugs, supplies and laboratory increased the ICERs 1.6 to 2 times higher as compared with the base-case. Despite such variations, all the interventions except calcium supplementation, remain at an ICER <US$500 per DALY averted. The most important cost driver for calcium supplementation was the drug cost, which accounted to 77% of the intervention’s total cost. Figure 1 shows the result of probabilistic sensitivity analysis. It illustrates the considerable uncertainty surrounding our cost-effectiveness estimates, with wide and overlapping cost and effectiveness ranges.

**Table 5:** ICERs for maternal and neonatal interventions under multiple scenarios

No.	Intervention	Base-case	Undiscounted health benefits	75% adherence to treatment	Double cost	50% effectiveness
1	Induction of labour (beyond 41 weeks)	152	76	229	256	306
2	Kangaroo mother care	8	4	12	12	16
3	Neonatal resuscitation (institutional)	7	3	10	11	13
4	Management of pre-eclampsia and eclampsia	108	65	162	189	217
5	Antibiotics for preterm pre-labour rupture of membrane (pPRoM)	69	35	104	124	139
6	Safe abortion	108	70	162	197	216
7	Antenatal corticosteroids for preterm labour	98	49	147	181	196
8	Newborn sepsis—injectable antibiotics	17	9	26	32	35
9	Maternal sepsis case management	220	133	330	415	440
10	Syphilis detection and treatment (pregnant women)	224	107	336	430	449
11	Active management of the third stage of labour	245	148	367	470	489
12	Tetanus toxoid (pregnant women)	168	84	252	328	336
13	Calcium supplementation	3081	1925	4770	6087	6167

## Discussion

Our analysis showed that 12 out of the 13 MNH interventions were highly cost-effective. All interventions that are included in our analysis (except calcium supplementation that is not yet implemented in public health facilities) are not entirely new to the Ethiopian health system. However, most of the interventions have a very low coverage rate. Therefore, assessing the cost-effectiveness of interventions that are currently implemented as well as adding those that could potentially be introduced can assist policymakers and planners in Ethiopia to prioritize the unfinished agenda of MNH. All interventions, besides calcium supplementation, were found to be very cost-effective (<US$300 per DALY) in Ethiopia. Adam *et al.* (2005) have reported a similar pattern to what we found in their CEA of strategies for MNH in developing countries (calcium supplementation and safe abortion were not included in their analysis however). A study conducted on the cost-effectiveness of calcium supplementation in Colombia concluded that varying the cost of calcium tablets or the incidence of pre-eclampsia renders the intervention no longer cost-effective for a threshold of three times Colombia’s GDP per capita (Becerra *et al.*, 2012).

Our results demonstrate that the interventions that could be delivered at the primary health care (PHC) level were very cost-effective. In the last decade, Ethiopia has introduced a health extension programme with the training and deployment of >38 000 health extension workers (HEWs) which represents an opportunity to scale-up MNH interventions at the community level (Federal Democratic Republic of Ethiopia Ministry of Health 2015; Federal Ministry of Health of Ethiopia 2007). HEWs could indeed play important roles such as identifying pregnant women and teaching them to identify complications that arise during pregnancy and responding immediately; or providing post-delivery follow-up care for the mother infant pair. Management of neonatal infection with antibiotics and community care of newborns were found to be effective in reducing neonatal mortality and scalable at the community level (Baqui *et al.*, 2009; Traa *et al.*, 2010). Furthermore, a programme intervention study conducted in the Southern part of Ethiopia (using a ‘continuum of care’ approach) to evaluate the delivery of essential antenatal and obstetric services in communities through HEWs resulted in a significant decline in both maternal deaths and stillbirths highlighting the feasibility and effectiveness of maternal and neonatal interventions in Ethiopia (Lindtjørn *et al.*, 2017, 2018). Delivery of services at the community level using HEWs has the additional benefit of bringing care to all women and infants, particularly to those socio-economically disadvantaged and marginalized rural residents. In our analysis, kangaroo mother care that was offered in neonatal facilities was very cost-effective (US$8 per DALY averted). Perhaps the introduction of KMC along with breastfeeding support for preterm/low birth weight newborns at the community level may even be a more cost-effective alternative than providing this service in neonatal wards.

Safe abortion care is one of the cost-effective services that can be delivered effectively at PHC facilities. The cost of treatment from the provider perspective to provide safe abortion using the medical method (vaginal misoprostol) for a single case was US$10 in Ghana, which is comparable to our estimate of an average cost per patient close to US$8 (Hu *et al.*, 2010). Safe abortion at the health centre level, such as manual vacuum aspiration or medical abortion using misoprostol resulted in substantial cost savings as compared with dilatation and curettage that is often hospital based. Most public health centres in Ethiopia are not currently providing safe abortion services (Federal Democratic Republic of Ethiopia Ministry of Health, 2015). The broadening of legal indications for abortion (the 2005 revised family law of Ethiopia) and the issuance of safe abortion technical guideline in 2006 created a favourable environment to scale-up delivery of safe abortion services in Ethiopia.

Despite the fact that most of the interventions delivered at the community level and in PHC facilities are very cost-effective, prevention of most maternal and neonatal deaths requires access to quality clinical care services. Improving quality of care is often considered very costly. However, a research project by the prevention of maternal mortality network in West Africa found that renovation or upgrading of essential obstetric care services in district hospitals and health centres was not as expensive as often assumed. Most developing countries have extensive health systems that are often under-utilized. With inputs such as opening operating rooms with a supply of electricity and blood banks, for less than $15 000, improvement in the provision of quality delivery care services with significant impact on maternal mortality have been seen (Prevention of Maternal Mortality Network, 1997). Ethiopia has undertaken an accelerated expansion of PHC facilities since 2003 and currently there are >3300 functional health centres that could serve as important inputs in the scale-up of obstetric care in Ethiopia (Federal Democratic Republic of Ethiopia Ministry of Health, 2015). Additionally, delivery of quality obstetric and neonatal services requires a reliable supply of medicines, functioning equipment and respectful provider attitude (Kruk *et al.*, 2010). Cultural factors also influence utilization of facility delivery care services. According to 2014 Ethiopian Mini DHS, 34% of rural women reported that facility deliveries were not customary highlighting the need for enhanced community mobilization (Central Statistical Agency, 2014).

The study has several limitations. First, our analysis does not include all possible maternal and neonatal interventions that could be considered in Ethiopia. Additional analysis may therefore be warranted. Second, the efficacy data we used were derived from studies conducted in more developed countries with higher quality of services and may not translate directly to Ethiopia. In the analysis of cost, we have included patient- and programme-level intervention costs that are incremental to the current coverage levels and fail to address the cost required for facility expansion. Data on perinatal mortality were not included in the analysis that may result in under estimation of the cost-effectiveness of interventions such as syphilis case detection and treatment. Due to the possibility of interactions in both cost and health impacts of implementing several single interventions at once; it is likely that the deaths averted could have been overestimated.

Despite these limitations, our findings can guide future health sector planning in Ethiopia. Along with the government’s vision to become a middle-income country, the Ministry of Health of Ethiopia has set ambitious health targets (Admasu *et al.*, 2014). The key strategy is ensuring universal access of basic health interventions for all Ethiopians mainly through strengthening PHC. The base-case scenario targets in Ethiopia for 2025 includes; a maternal mortality ratio of 260 per 100 000 live births, neonatal mortality rate of 28 per 1000 live births and 77% coverage for four antenatal care visits and skilled birth attendance. Even though our analysis considered a 20% point increase in coverage in a year, further scale-up of all individual interventions (excluding calcium supplementation) to the target coverage rates as set in the envisioning document will likely contribute to the achievement of maternal and neonatal mortality targets. The annual budget required to implement a 20% increase in coverage of all the 12 individual interventions costs an additional 0.11 US$per capita which is 0.46% of the 2013/14 annual total health expenditure (2.52 billion US$) for Ethiopia. In comparison, according to a study on CEA of cardiovascular diseases interventions in Ethiopia, combination of drug treatment for absolute risk of cardiovascular diseases >35% (which had the lowest ICER, 67 US$per DALYs averted) was estimated to cost 0.4% of the 2010/11 annual total health expenditure (1.6 billion US$) and averts about 107 000 DALYs (Tolla *et al.*, 2016). Similarly, a CEA of mental health interventions showed a 2.1% increase in annual total health budget (2012) would be required to implement the mental health interventions in Ethiopia with an expected health gain of 197 000 healthy life years (Strand *et al.*, 2015).

While evidence on cost-effectiveness is an important tool in prioritizing health interventions, it should not be the only consideration in the selection of interventions for implementation in a country. The priority setting process should also consider other socially desirable goals and major health system objectives such as equity and financial risk protection (World Health Organization, 2014). For example, most interventions included in our analysis could be further evaluated in terms of the gains in financial risk protection they bring to Ethiopian families (e.g. reduction in medical impoverishment related to maternal and child health conditions) (Johansson *et al.*, 2015; Pecenka *et al.* 2015; Verguet *et al.*, 2016; Memirie *et al.*, 2017). In this respect, many of the interventions examined in this article could be delivered at PHC facilities bringing care nearer to rural women and infants where most of the poorer Ethiopians reside (Moges, 2013).

## Conclusions

Given the substantial health dividend from investing in universal coverage of the intervention included in our analysis, this evidence could inform national policymakers to prioritize scale-up of maternal and child health interventions in Ethiopia. It can also inform the ongoing essential health services package revision in Ethiopia. Our analysis also suggests that calcium supplementation may not be prioritized for inclusion in Ethiopia’s essential MNH intervention package.

## Supplementary Material

czz034_Supplementary_Data

## Footnotes

1 Even though the management of pre-eclampsia/eclampsia could start during pregnancy it will usually continue during labour and delivery.
