# Supplementary material for: A cost-effectiveness analysis of maternal and neonatal health interventions in Ethiopia
Source: Health Policy Plan. 2019 May 18;34(4):289–97. doi: 10.1093/heapol/czz034 (PMC6661540; doi:10.1093/heapol/czz034)
Supplement: czz034_Supplementary_Data [file czz034_supplementary_data.docx]

**Supplementary data-Cost computation for maternal and neonatal health interventions in Ethiopia**

Cost is computed from the provider’s perspective. We first computed patient-level cost for the 13 individual maternal and neonatal health interventions included in our analysis. Based on a previous study in Ethiopia, we estimated program-level cost at 10% of patient-level cost (Mathewos et al. 2017). We used the same program–level cost across interventions, which is 10% of the average patient-level cost of all interventions in our study. Total intervention cost was the sum of patient- and program-level costs.

In the following section, we present the details of patient-level cost computations for each of the 13 interventions.

**Cost inputs**

Total number of live births in 2018 in Ethiopia (A) is 3,340338 (The Government of Federal Democratic Republic of Ethiopia 2017).

Number of unsafe abortion as a ratio of live births (B) is 20% (World Health Organization 2011).

Number of cases with preterm births as a ratio to live births (C) is 10.1% (Lee et al. 2013).

- Number of low birth weight babies requiring kangaroo mother care as a ratio of live births (C_1_) is 15%.

Number of cases with preterm pre-labor rupture of membrane births as a ratio to live births (D) is 4% (a third of the preterm births) (Cousens et al. 2010).

Number of cases with requiring induction of labor (>41 weeks of gestation) as a ratio to live births (E) is 5% (Avenir Health 2013).

Number of cases with pre-eclampsia/eclampsia as a ratio to live births (F) is 2.8% (Avenir Health 2013).

Number of cases with severe pre-eclampsia/eclampsia as a ratio to live births (G) is 1% (Avenir Health 2013).

Number of cases requiring treatment for neonatal sepsis as a ratio to live births (H) is 10% (Avenir Health 2013).

- 90% treated with injectable antibiotics (H_1_)
- 10% require full supportive care (H_2_)

Number of cases requiring neonatal resuscitation as a ratio to live births (I) is 1% (Avenir Health 2013).

- 10% of the births require stimulation (I_1_)
- 1% require bag and mask ventilation (I_2_)

Number of women requiring treatment for maternal sepsis as a ratio to live births (J) is 4.1% (Avenir Health 2013)

Number of pregnant women with syphilis as a ratio to live births (K) is 1.2% (The Ethiopian Public Health Institute 2015).

Incremental coverage level (L) is 20%.

Number of facility visits (M)

Number of hospital bed days (N)

Cost per visit at health center (O)

Cost per visit at hospital (P)

Cost per bed day at hospital (Q)

Total drug cost per patient (R)

Total laboratory cost (S)

Transportation multiplier (T)

**Details of drugs and supplies and laboratory test of each intervention**

| No. | Intervention | Drugs | Other supplies | Laboratory test |
| --- | --- | --- | --- | --- |
| 1 | Safe abortion | 800 μg vaginal misoprostol  Paracetamol 500mg twice per day for 3 days. | None | None |
| 2 | Tetanus toxoid | Two tetanus toxoid immunizations^#^ | None | None |
| 3 | Calcium supplementation | Calcium lactate 1200mg daily for 7 months | None | None |
| 4a | Management of pre-eclampsia (2.8% of all births) | Sodium lactate 500ml^#^  Hydralazine 20mg ampoule^#^ | Foley catheter  Urine bag (2L) | None |
| 4b | Management of severe pre-eclamsia/eclampsia (1% of all births) | Initial dose of MgSO4 (20%) 4g IV then IM injection 5gram of MgSO4 (50%) every 4 hours until delivery (1.5 days i.e. 9 injections assumed)^#^  Sodium lactate 500ml (4 bags)^#^  Lidocaine 2ml (2 ampoules) |  |  |
| 5 | Antibiotics for preterm pre-labor rupture of membrane | Erythromycin 250mg PO QID (four times per day) for 7days, Amoxicillin 500mg TID (three times per day) for 7days. | None | None |
| 6 | Antenatal corticosteroid for preterm labor | Betamethasone 12mg BID (twice per day) 24hours apart^#^ | None | None |
| 7 | Active management of third stage of labor | Oxytocin 10 International Unit, intramuscular injection^#^ | None | None |
| 8 | Induction of labor | Misoprostol 200mcg |  |  |
| 9 | Maternal sepsis case management | Ampicillin 2g QID IV, Gentamicin 240 mg (maximum) per day and Metronidazole 500mg IV TID for 04 days^#^  IV fluids: 4 litre of Normal Saline^#^  Amoxicillin 500mg po TID for 03 days  Paracetamol 1gm QID for 02 days | Foley catheter  Urine bag (2L) | Complete blood count |
| 10 | Newborn sepsis | 90% of the cases will receive injectable antibiotics for 07 days (Intramuscular injection of ampicillin and gentamicin)^#^ | None |  |
|  |  | 10% require full supportive care at a hospital for 10 days.  Sodium lactate 500ml (10 bags)^#^  Intravenous ampicillin 75mg TID and Gentamicin 10mg BID^#^  Oxygen therapy |  | Blood culture,  Cerebrospanial fluid analysis and culture |
| 11 | Syphilis case detection and treatment in pregnant women | Benzanthine penicillin 2.4 million unit^#^ | None | Rapid plasma reagin |
| 12 | Neonatal resuscitation | None | Bag and mask^±^ | None |
| 13 | Kangaroo mother care | None | None | None |
| ^#^Total drug costs for these interventions include costs of intramuscular or intravenous administration and cost to secure intravenous lines. ^±^Includes the cost of cleaning the bag after use (World Health Organization 2018). One bag and mask will be used to resuscitate an average of 20 cases. | | | | |

**Intervention cost computation**

1. Safe abortion

- Health service delivery cost = A*B*L*M*O
- Drug cost= A*B*L*R*T
- Total cost for safe abortion= Health service delivery cost + Drug cost + Program cost

1. Tetanus toxoid

- Health service delivery cost = A*L*M*O
- Drug cost= A*L*R*T
- Total cost for tetanus toxoid= Health service delivery cost + Drug cost + Program cost

1. Syphilis detection and treatment for pregnant women

- Health service delivery cost = A*L*M*O
- Drug cost= A*L*R*K*T
- Laboratory cost=A*L*S*T
- Total cost for syphilis detection and treatment= Health service delivery cost + Drug cost + Laboratory cost + Program cost

1. Management of pre-eclampsia/eclampsia

- Health service delivery cost = (A*F*L*M*P) + (A*G*L*N*Q)
- Drug cost= (A*F*L*R*T) + (A*G*L*R*T)
- Total cost for pre-eclampsia/eclampsia= Health service delivery cost + Drug cost + Program cost

1. Neonatal sepsis

- Health service delivery cost = (A*H_1_*L*M*O) + (A*H_2_*L*N*Q)
- Drug cost= (A*H_1_*L*R*T) + (A*H_2_*L*R*T)
- Laboratory cost=A*H_2_*L*S*T
- Total cost for neonatal sepsis= Health service delivery cost + Drug cost + Laboratory cost + Program cost

1. Neonatal asphyxia

- Health service delivery cost = (A*I_1_*70%*L*M*O) + (A*I_1_*30%*L*M*P)
- Drug cost= (A*I_2_*L*R*T)
- Total cost for neonatal asphyxia= Health service delivery cost + Drug cost + Program cost

1. Maternal sepsis

- Health service delivery cost = (A*J*L*N*Q)
- Drug cost= (A*J*L*R*T)
- Laboratory cost=A*J*L*S*T
- Total cost for maternal sepsis= Health service delivery cost + Drug cost + Laboratory cost + Program cost

1. Kangaroo mother care

- Health service delivery cost = (A*C_1_*70%*L*M*O) + (A*C_1_*30%*L*M*P)
- Total cost for kangaroo mother care= Health service delivery cost + Program cost

1. Induction of labor

- Health service delivery cost = (A*F*L*M*P)
- Drug cost= (A*F*L*R*T) + (A*G*L*R*T)
- Total cost for induction of labor= Health service delivery cost + Drug cost + Program cost

1. Antibiotics for preterm pre-labor rupture of membranes

- Health service delivery cost = (A*D*L*N*Q)
- Drug cost= (A*D*L*R*T)
- Total cost for antibiotics for preterm pre-labor rupture of membranes= Health service delivery cost + Drug cost + Program cost

1. Antenatal corticosteroids for preterm labor

- Health service delivery cost = (A*C*L*N*Q)
- Drug cost= (A*C*L*R*T)
- Total cost for antenatal corticosteroids for preterm labor = Health service delivery cost + Drug cost + Program cost

1. Active management of third stage of labor

- Health service delivery cost = (A*70%*L*M*O) + (A*30%*L*M*P)
- Drug cost= A*L*R*T
- Total cost for active management of third stage of labor= Health service delivery cost + Drug cost + Program cost

1. Calcium supplementation

- Health service delivery cost = (A*L*M*O)
- Drug cost= A*L*R*T
- Total cost for calcium supplementation^[[1]](#footnote-1)^= Health service delivery cost + Drug cost + Program cost

References

1. Mathewos B, Owen H, Sitrin D, et al. 2017. Community-Based Interventions for Newborns in Ethiopia (COMBINE): Cost-effectiveness analysis. Health Policy and Planning 32:i21-i32.
2. The Government of Federal Democratic Republic of Ethiopia. 2017. Proposal for support submitted to the Global Alliance for Vaccines and Immunization (GAVI) and The Vaccine Fund. Addis Ababa; Ethiopia.
3. World Health Organization (WHO). 2011. Unsafe Abortion: Global and Regional Estimates of the Incidence of Unsafe Abortion and Associated Mortality in 2008, sixth ed., Geneva: WHO.
4. Lee AC, Katz J, Blencowe H, et al. 2013. National and regional estimates of term and preterm babies born small for gestational age in 138 low-income and middle-income countries in 2010. The Lancet Global Health 1:e26-36.
5. Cousens S, Blencowe H, Gravett M, Lawn JE. 2010. Antibiotics for pre-term pre-labor rupture of membranes: prevention of neonatal deaths due to complications of pre-term birth and infection. International Journal of Epidemiology 39 Suppl 1:i134-i143.
6. Avenir Health. 2013. OneHealth Model, intervention treatment assumptions.
7. The Ethiopian Public Health Institute. 2015. Report on the 2014 round antenatal care based sentinel HIV surveillance in Ethiopia. Addis Ababa, Ethiopia.
8. World Health Organization. 2018. Health services delivery costs. <http://www.who.int/choice/cost-effectiveness/inputs/health_service/en/>.
9. Hofmeyr GJ, Lawrie TA, Atallah ÁN, Duley L. 2010. Calcium supplementation during pregnancy for preventing hypertensive disorders and related problems. Cochrane Database of Systematic Reviews, Issue 8. Art. No.: CD001059. DOI:10.1002/14651858.CD001059.pub3.

1. Calcium supplementation results in a 50% reduction in pre-eclampsia cases (Hofmeyr et al. 2010). The cost saving that results from prevention has been deducted from the total cost in the computation of cost-effectiveness analysis. [↑](#footnote-ref-1)
